# A simple method for quick evaluation of the anterior tooth ratio: an observational study

**DOI:** 10.1186/s12903-022-02517-3

**Published:** 2022-11-16

**Authors:** Guanning Zhang, Qingchen Feng, Xiaoyu Wei, Ke Xing, Hongxiang Mei, Jiawei Zhou, Chen Jiang, Juan Li

**Affiliations:** 1 Department of Orthodontics, West China Hospital of Stomatology, Sichuan University, 14#, 3rd Section, Renmin South Road, Chengdu, 610041 China; 2 State Key Laboratory of Oral Diseases, National Clinical Research Center for Oral Diseases, West China Hospital of Stomatology, Sichuan University, 14#, 3rd Section, Renmin South Road, Chengdu, 610041 China

**Keywords:** Anterior tooth ratio, Tooth size, Dental cast analysis, Diagnosis and treatment planning

## Abstract

**Background:**

An ideal relationship of anterior teeth is closely related to postoperative function, stability, and aesthetics. Therefore, it is necessary to estimate the proportion of anterior teeth when communicating with patients about possible treatment plans and outcomes. This study aimed to establish a simple method for assessing the proportion of anterior teeth and to identify the standard ratio value to provide references for clinical work.

**Methods:**

Five hundred fourteen patients were divided into derivation, standard, and validation datasets. We first deduced our novel simplified anterior tooth ratio (SATR) by finding the key teeth with the derivation datasets, then established standard values by measuring the standard models, and finally validated the diagnostic performance of SATR. Independent sample t-test was used to select key teeth. Pearson’s correlation analysis and linear regression analysis was used to test and verify the correlation between SATR and the anterior Bolton ratio. Chi-square test and diagnostic test were used to verify the diagnostic results using SATR. *P* values of < 0.05 were considered statistically significant.

**Results:**

Patients with an abnormal anterior Bolton ratio were more likely to have variations in the maxillary and mandibular lateral incisors. Therefore, the ratio of maxillary and mandibular lateral incisors was chosen as a simple way to assess the anterior tooth ratio and was defined as SATR (simplified anterior tooth ratio). A positive correlation was observed between SATR and anterior Bolton ratio (r = 0.702, *p* < 0.001), with the linear regression equation as follows: y = 0.503 + 0.328x, x = SATR, y = anterior Bolton ratio. The standard value of SATR was established (85.69% ± 3.57%) and proven reliable in clinical practice.

**Conclusions:**

The ratio of maxillary and mandibular lateral incisors can be used to estimate the anterior tooth ratio, which showed high reliability and efficiency.

## Background

Aesthetic appearance influences social psychology and quality of life, and it has gradually become the main reason for people seeking orthodontic treatment [1]. One of the primary guidelines for orthodontic and dental treatment is the aesthetic consideration [2]. Different levels of aesthetic appearance are made up of macroaesthetics, microaesthetics, and miniaesthetics. Tooth size and morphology, tooth ratio, and tooth symmetry, not only influence tooth alignment and occlusal relationship, but are also linked to microaesthetics [3, 4]. According to studies, patients have a sharper perception of the asymmetry of the upper central incisors, confirming that the symmetry of the dentition will directly affect the patient’s evaluation of smile aesthetics [5]. Some studies have also confirmed that the ratio of upper and lower anterior teeth affects the overbite and overjet of anterior teeth, thereby influencing the aesthetics of patients’ dentition and face, which is also accompanied by functional influences such as the establishment of normal anterior guidance, stomatognathic system function, temporomandibular joint health, and the maintenance of orthodontic efficacy [6–8]. Therefore, the ratio of upper and lower anterior teeth has always been the focus of orthodontists as an important component of microesthetic analysis and function establishment.

Wayne Bolton [9] first presented the idea that a certain ratio of maxillary to mandibular tooth size is important in the formation of proper occlusion. He measured the mesiodistal width of maxillary and mandibular teeth in models of 55 white female with excellent occlusions and obtained the ideal values of anterior tooth ratio and overall tooth ratio. Little’s irregularity index (LII) aims to objectively quantify the misalignment of mandibular anterior teeth [10], which is gradually used in the analysis of irregularity of upper and lower teeth and the stability of curative effect now [11, 12]. However, some studies have confirmed the low repeatability and reliability of LII measurement [13, 14]. The Bolton analysis is now commonly used by clinicians as an objective method for the precise measurement of tooth proportion to guide clinical diagnosis and treatment. This has been studied across different ethnicities and regions [15–19], among various types of malocclusion [20–24], and in the presence of new indicators [25]. These studies did improve the accuracy and applicability of the Bolton analysis. However, when a patient is first admitted to the clinic and ask for the treatment methods and efficacy, the Bolton analysis may not be practical to perform. In such situations, there is a need for a simple method that allows for the quick assessment of anterior tooth ratio, which can potentially supplement the Bolton analysis and allow clinicians to offer timely suggestions. It can also reduce doctor-patient disputes regarding treatment methods that may cause tooth damage or yield imperfect results.

The variation of upper lateral incisors is one of the main causes of tooth-size discrepancy [17]. Al-Abdallah [26] found that microdontia of the upper lateral incisors was the only dental anomaly with a significantly increased prevalence among patients with maxillary versus mandibular hypodontia (46.7%). Furthermore, patients with upper lateral incisors agenesis had an increased prevalence rate of permanent tooth agenesis (18.2%) according to Garib [27]. The high incidence of upper lateral incisor malformation indicates that the width of upper lateral incisors is a key factor that affects tooth-size discrepancy and malocclusion.

Our study aims to selected the key teeth that changed alongside the anterior Bolton ratio to establish a simple method for the rapid screening of anterior tooth ratio. We provided a standard value for orthodontists and confirmed its diagnostic efficiency.

## Methods

### Samples

Ethical approval was obtained from the ethics committee of. Patients who received treatment at the Department of Orthodontics,, were selected for the study. All patients were of Han nationality, born and living in China.

#### Derivation datasets

For the derivation datasets, 240 patients (65 men and 175 women) with an average age of 24.90 ± 7.89 years (11–51 years) were enrolled.

The inclusion criteria were as follows:

1. Permanent dentition: complete eruption of teeth except second and third molars;
2. High quality oral scan models: complete crown surface, no missing anatomical landmarks, no blurring or ghosting;
3. Complete dentition: no missing teeth, no supernumerary teeth, no retained deciduous teeth; and
4. Normal tooth morphology: no malformed teeth such as peg-shaped teeth (the incisal mesiodistal width of the tooth crown is shorter than the cervical width [28]) and fused teeth.

The exclusion criteria were as follows:

1. Damage affecting the adjacent surface of teeth: obvious abrasion, extensive caries, and defects;
2. Restorations;
3. History of interproximal enamel reduction;
4. Jaw deformity requiring orthognathic surgery, cleft lip and palate, any craniomaxillofacial syndrome;
5. Systemic diseases.

#### Standard datasets

For the standard datasets, 132 patients (37 men and 95 women) with an average age of 25.17 ± 7.24 years (11–43 years) were enrolled. The inclusion and exclusion criteria for this part were the same as in 2.1.1, except that perfect occlusion after treatment was required.

The definition of perfect occlusion was as follows:

1. Criteria for anterior teeth:
  Neutral inter-arch canine relationship: the cusp of the maxillary canine bite between the cusp of the mandibular canine and buccal-cusp of the first premolar and the mesial-distal deviation is less than 0.5 mm.
  Normal mesiodistal crown angulation: the values of the crown angle are all positive.
  Normal buccolingual inclination of tooth long axis: evaluated by the cephalometric, U1-SN = 105.7° ± 6.3° and L1-MP = 96.5° ± 7.1°.
  Normal overbite and overjet: the incisal edge of the upper incisors covers the upper 1/3 of the crown of the lower incisors, and the lingual margin of the incisal edge of the upper incisors covers the labial surface of the crown of the lower incisor by 2 ± 1 mm. Overbite was measured vertically in the occlusal plane, whereas overjet was measured horizontally in the occlusal plane.
  Aligned midline of dentition.
  No rotation, crowding, and space with normal proximal contacts.
2. Criteria for posterior teeth:
  The teeth are aligned, without obvious dislocation, rotation, crowding and space, and without abnormal mesiodistal inclination and buccolingual inclination.

#### Validation datasets

For the validation datasets, 142 patients (41 men and 101 women) with an average age of 25.02 ± 7.88 years (11–50 years) were enrolled to undergo diagnostic tests. This part of the dataset came from two dental clinics in Chengdu within 4 months after the establishment of the standard value. The inclusion and exclusion criteria for this part were the same as in 2.1.1.

### Processes

**Fig. 1:**
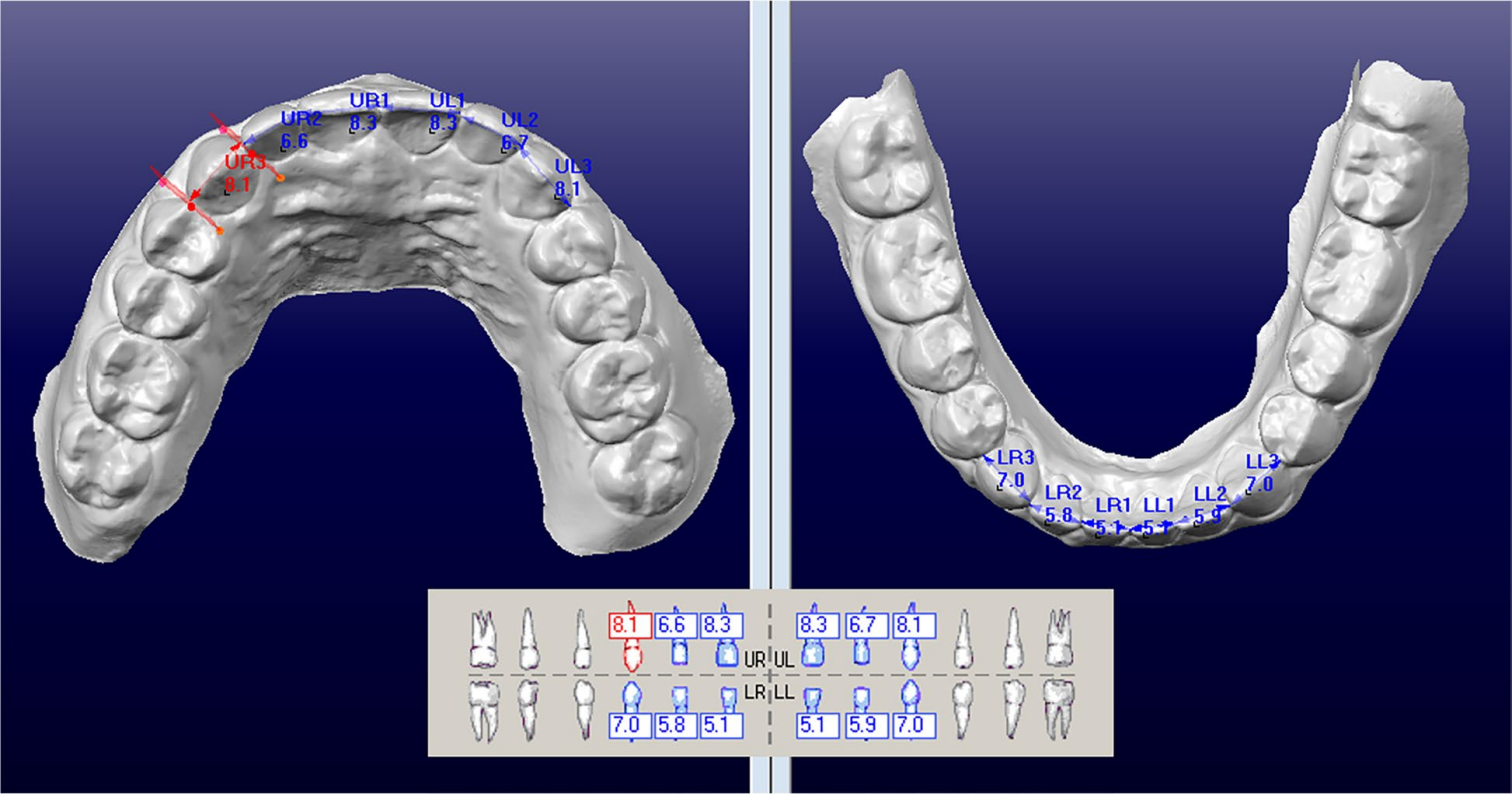
Measurement of the anterior Bolton ratio using the OrthoCAD 5.9.0

The oral scan data of all samples obtained using the iTero® Element 2 (Align Technology, San Jose, Calif) were exported to the Rhinoceros file format (*.3 dm) and imported into the OrthoCAD software (version 5.9.1, Calif Cadent, Carlstadt, NJ) to generate 3D digital models. Operators G.Z. and K.X., who have received 2 years of clinical training in orthodontics, performed measurements using the OrthoCAD software under the guidance of Dr. Li, an orthodontist with over 20 years of experience. After 2 weeks, 20% of the total sample was selected randomly and then remeasured by the same operators to test the error of the method. The width of the anterior dental crown was defined as the greatest interproximal distance from the mesial contact point to the distal contact point, wherein 12 anterior teeth from the upper and lower dentition were measured to calculate the anterior Bolton ratio (Fig. 1).

The dental plaster model was turned into a digital model via laser scanning and then imported into the OrthoCAD 5.9.0 software. We used the Bolton measurement module to fix a point after three-dimensional observation and measured the widths of upper and lower anterior teeth.

#### Select key teeth of SATR (simplified anterior tooth ratio)

From the derivation datasets, 240 samples were divided according to the value of the anterior Bolton ratio, into the low (< 77.08%), normal (77.08–80.52%), and high value (> 80.52%) groups. We calculated the average width of the crown of bilateral central incisors, lateral incisors, and canines across the three groups. Then, one was selected from each dental arch that had statistical differences among all the three groups as the key teeth.

#### SATR formula and correlation with the anterior Bolton ratio

The simplified formula was built as follows: SATR% = (Sum of width of lower key teeth)/(Sum of width of upper key teeth) %. Afterward, the correlation between SATR and the anterior Bolton ratio was evaluated by calculating the correlation coefficient. Finally, an appropriate linear regression equation was established.

#### Standard value of SATR

After confirming the correlation, we measured the width of upper and lower key teeth of the 132 posttreatment models from standard datasets with perfect occlusion. These measurements were plugged into the simplified equation to establish the standard value of SATR. The resulting value was plugged back into the linear regression equation obtained in 2.2.2 to test whether it agrees with the standard value of anterior Bolton ratio.

#### Clinical study for the diagnostic reliability of SATR

New patients were enrolled as validation samples in clinical work within 4 months after the establishment of the standard value, and diagnostic tests were performed. The anterior Bolton ratio was used as the gold standard for categorizing anterior tooth ratios as either deficient (< 77.08%), normal (77.08–80.52%), or excess (> 80.52%). SATR was also measured in these patients to identify deficient (< 82.12%), normal (82.12–89.26%, and excess (> 89.26%) anterior tooth ratios. The consistency (determined by the χ^2^ value), sensitivity, specificity, positive predictive value (PPV), and negative predictive value (NPV) of SATR were obtained.

### Statistical analysis

Data were analyzed using the SPSS software (version 22.0; SPSS, Chicago, III). ICC (intraclass correlation coefficient) was used to assess the intraclass and interclass consistency. Independent sample t-test was used to select key teeth. Pearson’s correlation analysis was used to test and verify the correlation between SATR and the anterior Bolton ratio. Linear regression analysis was used to establish the linear regression equation based on SATR. Chi-square test and diagnostic test were used to test the consistency between diagnostic results using SATR versus the anterior Bolton ratio. *P* values of < 0.05 were considered statistically significant. If the data were found to have normal distribution (i.e., Kolmogorov–Smirnov test, *P* > 0.05) and homogeneity of variance (Levene’s test, P > 0.05), the parametric test method was used to evaluate the data. Otherwise, the nonparametric test method was used.

## Results

The results of ICC of the intraclass and the interclass consistency were 0.954–0.986 and 0.956–0.990, respectively, signifying good reliability and reproducibility of the model measurement.

### Patients with an abnormal anterior Bolton ratio were more likely to have variation in the width of upper and lower lateral incisors

In the normal value group, the average width of upper central incisors, lateral incisors, and canines were 8.650 ± 0.490 mm, 7.216 ± 0.511 mm, and 8.007 ± 0.522 mm, respectively, whereas that of the lower central incisors, lateral incisors, and canines were 5.654 ± 0.418 mm, 6.172 ± 0.350 mm, and 6.961 ± 0.531 mm, respectively. Compared with the normal value group, both the low and high value groups had significantly different upper and lower lateral incisor width (*P* < 0.05, Table 1), whereas the widths of the other anterior teeth were not significantly different (*P* > 0.05, Table 1). Therefore, the upper and lower lateral incisors were confirmed to be the key teeth that influence the anterior Bolton ratio; these were selected as the key teeth of SATR.

**Table 1:** Discrepancies in upper and lower lateral incisors width of different anterior Bolton ratio (ABR)

		Mean Value(mm)	t	P
Width of upper central incisors	Normal ABR	8.650 ± 0.490	−1.750	0.082
	Small ABR	8.768 ± 0.409		
	Normal ABR	8.650 ± 0.490	1.784	0.076
	Large ABR	8.483 ± 0.448		
	Small ABR	8.768 ± 0.409	3.229	0.002^**^
	Large ABR	8.483 ± 0.448		
Width of upper lateral incisors	Normal ABR	7.216 ± 0.511	−2.436	0.016^*^
	Small ABR	7.387 ± 0.424		
	Normal ABR	7.216 ± 0.511	2.654	0.009^**^
	Large ABR	6.957 ± 0.468		
	Small ABR	7.387 ± 0.424	4.686	0.000^**^
	Large ABR	6.957 ± 0.468		
Width of upper canines	Normal ABR	8.007 ± 0.522	−1.387	0.167
	Small ABR	8.106 ± 0.424		
	Normal ABR	8.007 ± 0.522	1.111	0.268
	Large ABR	7.897 ± 0.458		
	Small ABR	8.106 ± 0.424	2.291	0.024^*^
	Large ABR	7.897 ± 0.458		
Width of lower central incisors	Normal ABR	5.654 ± 0.418	4.125	0.000^**^
	Small ABR	5.424 ± 0.314		
	Normal ABR	5.654 ± 0.418	−0.585	0.559
	Large ABR	5.700 ± 0.322		
	Small ABR	5.424 ± 0.314	−4.167	0.000^**^
	Large ABR	5.700 ± 0.322		
Width of lower lateral incisors	Normal ABR	6.172 ± 0.350	3.010	0.003^**^
	Small ABR	6.023 ± 0.320		
	Normal ABR	6.172 ± 0.350	−2.257	0.025^*^
	Large ABR	6.324 ± 0.329		
	Small ABR	6.023 ± 0.320	−4.437	0.000^**^
	Large ABR	6.324 ± 0.329		
Width of lower canines	Normal ABR	6.961 ± 0.531	0.663	0.508
	Small ABR	6.914 ± 0.401		
	Normal ABR	6.961 ± 0.531	−0.888	0.376
	Large ABR	7.047 ± 0.331		
	Small ABR	6.914 ± 0.401	−1.669	0.098
	Large ABR	7.047 ± 0.331		

ABR is short for anterior Bolton ratio

* Significant difference at level *P* < 0.05

** Significant difference at level *P* < 0.01

### The simplified formula of SATR strongly correlated with the anterior Bolton ratio

The simplified formula was determined as follows: SATR% = (Sum of width of lower lateral incisors)/(Sum of width of upper lateral incisors) %. The correlation analysis between SATR and the anterior Bolton ratio revealed a positive correlation with statistical significance (r = 0.702, *P* < 0.001, Table 2). Based on this, a linear regression equation was obtained: y = 0.503 + 0.328x (x = SATR, y = anterior Bolton ratio, *P* < 0.001).

**Table 2:** Correlation between SATR and anterior Bolton ratio

		Anterior Bolton ratio
SATR	Pearson	0.702
	P	0.000^**^

SATR is short for simplified anterior tooth ratio

** Significant difference at level *P* < 0.01

### Standard value of SATR

**Fig. 2:**
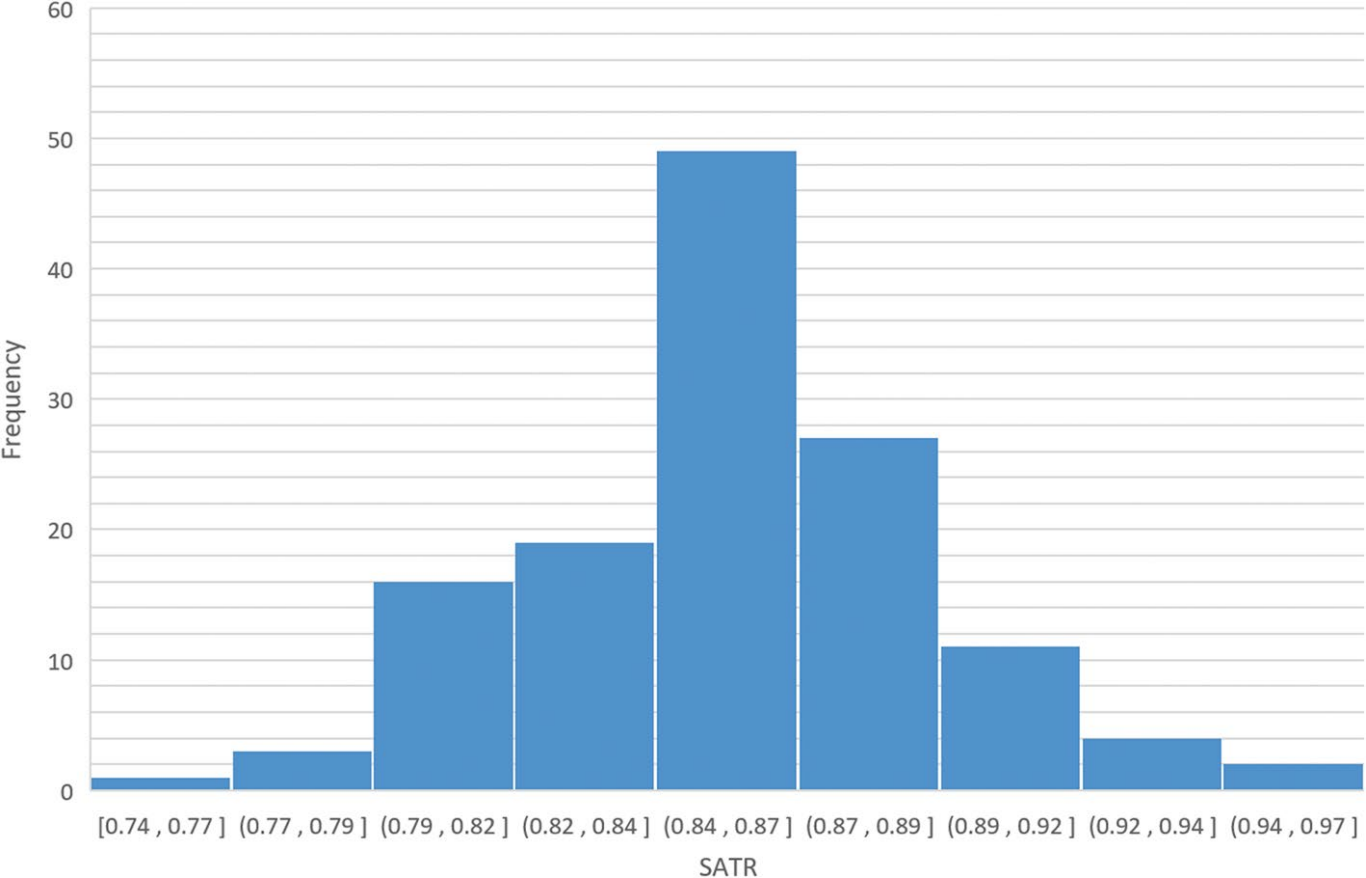
Simplified anterior tooth ratio (SATR) statistical distributions

Based on 132 models with perfect occlusion, the standard value of SATR was determined to be 85.69% ± 3.57% (Fig. 2, Table 3). After plugging this into the linear regression equation obtained in 3.2, this was found to be in accordance with the generally accepted normal values in the Chinese population (78.8% ± 1.72%).

**Table 3:** Descriptive statistics of SATR and the standard value

	Min.	Max.	Mean value	Std.
SATR	0.7441	0.9680	0.8569	0.0357

SATR is short for simplified anterior tooth ratio

The histogram was drawn according to the SATR value of 132 models with perfect occlusion.

### Reliability of the SATR in clinical work

The results of the two diagnostic methods are shown in Table 4. After calculation, χ^2^ = 2.59 and *P* = 0.27 (> 0.05) were obtained, signifying that the results of the two methods were consistent. Both deficiency and excess groups were included in the abnormal group, and the results of the two diagnostic methods are listed in Table 5. The statistical values of diagnostic tests listed in Table 6 indicated a good ability to judge the proportion of anterior teeth of SATR.

**Table 4:** Diagnosis (three classifications) of anterior Bolton ratio and SATR

Diagnostic test (SATR)	Gold standard diagnosis			Total
	Deficiency	Normal	Excess	
Deficiency	29	5	0	34
Normal	13	59	2	74
Excess	0	12	22	34
Total	42	76	24	142

SATR is short for simplified anterior tooth ratio

**Table 5:** Diagnosis (two classifications) of anterior Bolton ratio and SATR

Diagnostic test (SATR)	Gold standard diagnosis		Total
	Normal	Abnormal	
Normal	59	15	74
Abnormal	17	51	68
Total	76	66	142

Abnormal group included deficiency and excess groups

SATR is short for simplified anterior tooth ratio

**Table 6:** Statistical values for diagnostic tests

Sensitivity (Sen)	77.63%
Specificity (Spe)	77.27%
Positive predictive value (PPV)	79.73%
Negative predictive value (NPV)	75.00%

## Discussion

When seeing a patient for the first time, the orthodontists may need to make a quick assessment based on the patient’s oral conditions, including the ratio of the anterior teeth to answer the patient’s questions regarding treatment methods and expected outcomes. This is most commonly achieved using the Bolton analysis, which involves measuring the size of 12 anterior teeth to calculate the anterior tooth ratio. This yields relatively accurate results, but it is not suited for rapid judgment and screening. Our study established a simple method for evaluating the anterior tooth ratio by selecting only 4 key teeth. This method was based on and compared with the Bolton analysis as the gold standard, with the added benefit of reflecting the tooth ratio of the same dentition. Moreover, we obtained the standard value of this new method and verified its diagnostic performance.

The oral scan model has the advantages of easy storage, easy extraction, and high visibility, and it frequently provides patients with a better experience in clinical diagnosis and treatment [29, 30]. Numerous studies have confirmed that measuring oral scan digital models, especially tooth width and Bolton ratio, has the same accuracy as traditional plaster model measurement [30–34]. Similarly, there is no statistical difference in the accuracy of the scanning models of oral scanners from different manufacturers [35, 36]. As a result, we ultimately choose iTero® scanner to obtain digital models, as well as perform model measurement and analysis on 3D software, which also complies with the digital process of dental diagnosis and treatment.

The first step of our research was to identify the key teeth that reflect the proportion of the anterior teeth to establish a simplified formula (i.e., the SATR). Morphological variations of the upper lateral incisors are common in clinical practice, and deformities of the upper incisors are especially frequent among patients with different types of malocclusions [26, 27]. Prosthetic restoration of the undersized maxillary lateral incisors has been proven to return the Bolton ratio to the normal range [37]. In contrast, morphological variations of upper central incisors and canines are relatively rare. There is no significant difference in the width of the left and right anterior teeth with the same name between genders [38–40], however, the incidence of asymmetrical lateral incisors may be relatively high [41]. Studies have shown that patients are less sensitive to the morphological asymmetry of upper lateral incisors than upper central incisors [5]. However, the morphological variation of central incisors is relatively rare, while the lateral incisors variation has a significant impact on the upper-lower tooth ratio. As a result, we took the average width of left and right lateral incisors to derive the SATR formula, and analyze the bilateral upper and lower anterior teeth as an aesthetic entirety instead of considering the aesthetic impact of a particular tooth. This can also avoid the variability in results caused by possible lateral incisors asymmetry. Due to the morphological characteristics potentially causing large errors in mesiodistal measurements, peg-shaped tooth samples were not included in this research. Nevertheless, our study showed that the width of upper lateral incisors had statistically significant differences in all three different values of groups of the anterior Bolton ratio, whereas the upper central incisors and canines showed statistical differences in only two of three groups. Interestingly, a similar pattern was seen in the lower anterior teeth. Thus, only the lateral incisors changed strictly with the Bolton ratio. In other words, variations of the upper and lower lateral incisors cause the most changes in anterior tooth ratio. Thus, orthodontists need to focus on the size of the upper and lower lateral incisors to clarify the underlying source of the anterior tooth ratio imbalance, aid in implementing treatment methods like interproximal enamel reduction, and predict efficacy.

The second step of our research was to preliminarily affirm the evaluation of the anterior tooth ratio was consistent between the SATR and anterior Bolton ratio. After that, we obtained the regression equation of the anterior Bolton ratio and SATR, suggesting that the SATR can be used to infer the anterior Bolton ratio and mutually verify the actual measured value.

The third step of the study was to formulate the standard value of SATR in accordance with characteristics of Chinese population, which can be used quickly and directly in clinical work. The differences in tooth size and Bolton ratio between people of different ethnicities and regions have become a consensus. A study by Lavelle [15] between Caucasians, Asians, and African Americans and concluded that African Americans had higher anterior and overall teeth ratios compared with the other two ethnicities. Likewise, Smith [17] also found that the overall ratio of the African Americans was larger than that of Caucasians, and this was attributed to the difference in the size of the posterior teeth. Significant differences were also seen in the anterior ratio or overall ratio compared with Bolton’s original ratios among certain populations, including those from India [18], Turkey [16], Iran [19], and Saudi Arabia [42]. As early as 1991, the Chinese have established their own standard value of the Bolton ratio [43], and since then, many have studied and established standard values in different regions of China. Since the standard value was established 30 years ago, we hoped to determine a new standard value using the SATR to provide a reference for analysis of the anterior tooth ratio today. As for gender, most studies of different races found no significant difference in the Bolton ratio between men and women populations [16, 18–20, 22]; thus, we did not consider gender differences when selecting the sample.

In the process of establishing the standard value of the SATR, we found that several posttreatment models with normal occlusion of canines and posterior teeth, as well as those with normal overbite and overjet of anterior teeth, showed a large offset in measurement when the SATR analysis method was applied. Besides errors of model analysis, the most probable cause was the evidently imbalanced size of the teeth in the same dental arch; this revealed the implications of SATR on the disproportions of teeth in the same dentition. Values close to the upper and lower limits of the standard value can possibly indicate an imbalance of the lateral incisors among those with a normal anterior Bolton ratio. For example, there may be cases wherein the results of the Bolton analysis signify that the anterior tooth ratio is appropriate, but the patient’s upper lateral incisors are too small and the upper central incisors and canines are too large, or the lower lateral incisors are too large and the lower central incisors and canines are too small. In these situations, there may be poor cosmetic outcomes due to the imbalanced proportion of the teeth in the same dentition. Therefore, we thought the SATR may play a complementary role to the Bolton ratio.

Bioesthetic dentistry is a conservative approach which aims to restoring teeth to their natural form and function. This concept, which requires the patient’s teeth, lips, smile and face to be examined and designed as a whole, is a kind of novel “trend” in today’s world and an aspect of dental treatment that needs to be focused [44]. A large number of studies and clinical practices tell us that patients pay more attention to the aesthetics of the anterior teeth, which has even exceeded the requirements for the aesthetics of the full dentition [45]. Our study confirmed that not only anterior teeth are important in determining tooth aesthetics, but also the proportion of anterior teeth is important in determining dentition proportion, and the morphological variation of lateral incisors is the most important determinant of the relationship between anterior teeth. We can correct the imbalance proportion of anterior teeth with interproximal enamel reduction (IPR), lateral incisors restoration, and posterior occlusal relationship compromise. When selecting a treatment option, bioesthetics and its related anterior teeth aesthetics should always be the most important consideration. For instance, using the golden ratio or the other suitable natural ratio when designing the width of the upper anterior teeth can help to create an attractive smile [4, 46]. In addition to the anterior tooth index, correct color assessment and materials selection are also important steps in the restoration treatment of anterior teeth [47]. For example, more precise optical devices for artificial tooth color selection can be used [48], and the aesthetic impact of aging restoration materials should be considered [49]. Wax-up and mock-up can be very useful tools in treatment planning because they combine patients’ expectations with medical possibilities by the visibility of design and efficacy [50].

We have begun to verify the diagnostic value of SATR clinically. Including samples from different clinics as validation datasets was aimed to confirm the spatiotemporal validity of the new diagnostic method. Considering that the diagnostic results include three types (deficient, normal and excess), we supplemented the chi-square for consistency test. Since both deficient and excess were diagnosed as abnormal in diagnostic tests, we did not list the results of accuracy to avoid confusion. Using the anterior Bolton ratio as the gold standard, the sensitivity, specificity, PPV, and NPV of SATR were all greater than 75%, which was relatively high. Since our validation datasets were collected within a small period, the sample size may not be sufficient. We hope to accumulate larger samples for continuous verification in the future.

Nevertheless, this study still has some limitations. The shape of upper lateral incisors, specifically those that were trapezoid-shaped (i.e., narrow incisal edge and wide cervical part), may affect the accuracy of the measurement. Furthermore, excessive thickness of the crown of the upper anterior teeth or the varying shape of canines may affect the examiner’s judgment of overbite and overjet of anterior teeth and the neutral occlusion relationship of canines. Future studies should include a larger sample size and increased training for examiners in recognizing variations in tooth morphology. This also reminds us that when there are obvious variations in tooth shape, the use of SATR may not be as accurate for inexperienced doctors. Thus, we will continue to include samples for verification and correction. In the future, we hope that researchers in other countries can develop standard SATR values for different ethnic groups, as well as verify and improve this method so that it is more useful in clinical practice.

## Conclusions

The ratio of maxillary and mandibular lateral incisors can be used as a simplified calculation of the anterior tooth ratio, which was proven to be effective and reliable. We also established its standard value as 85.69% ± 3.57% for clinical use.

## Data Availability

The datasets generated during and analyzed during the current study are not publicly available considering that individual privacy might be compromised, but are available from the corresponding author on reasonable request.

## Abbreviations

SATR: Simplified anterior tooth ratio
ABR: Anterior Bolton ratio
ICC: Intraclass correlation coefficient
PPV: Positive predictive value
NPV: Negative predictive value
